# Isolation and antibiotic susceptibility of *E. coli *from urinary tract infections in a tertiary care hospital

**Published:** 2014

**Authors:** Sumera Sabir, Aftab Ahmad Anjum, Tayyaba Ijaz, Muhammad Asad Ali, Muti ur Rehman Khan, Muhammad Nawaz

**Affiliations:** 1 Sumera Sabir, B.S (Microbiology), Department of Microbiology, Faculty of Veterinary Science, University of Veterinary and Animal Sciences, Lahore, Pakistan.; 2 Aftab Ahmad Anjum, PhD (Microbiology), Department of Microbiology, Faculty of Veterinary Science, University of Veterinary and Animal Sciences, Lahore, Pakistan.; 3 Tayyaba Ijaz, PhD (Microbiology), Microbiology Diagnostic and Research Lab, Mayo Hospital, King Edward Medical University, Lahore, Pakistan.; 4 Muhammad Asad Ali, M. Phil (Microbiology), Department of Microbiology, Faculty of Veterinary Science, University of Veterinary and Animal Sciences, Lahore, Pakistan.; 5 Muti ur Rehman Khan, PhD (Pathology), Department of Pathology, Faculty of Veterinary Science, University of Veterinary and Animal Sciences, Lahore, Pakistan.; 6 Muhammad Nawaz, PhD (Medical Microbiology), Department of Microbiology, Faculty of Veterinary Science, University of Veterinary and Animal Sciences, Lahore, Pakistan.

**Keywords:** Urinary tract infections, Prevalence, *E. coli*, Antibiotic resistance, MDR

## Abstract

***Objective:*** The study was conducted to isolate and determine the antibiotic resistance in *E. coli* from urinary tract infections in a tertiary care hospital, Lahore.

***Methods:*** Urine samples (n=500) were collected from patients with signs and symptoms of Urinary tract infections. Bacteria were isolated and identified by conventional biochemical profile. Antibiotic resistance pattern of *E. coli* against different antibiotic was determined by Kirby-Baur method.

***Results:*** Bacterial etiological agent was isolated from 402 samples with highest prevalence of *E. coli* (321, 80%) followed by *Staphylococcus aureus* (9.4%), *Proteus species (*5.4%) and *Pseudomonas species* (5.2%). The *E. coli* were highly resistant to penicillin (100%), amoxicillin (100%) and cefotaxime (89.7%), followed by intermediate level of resistance to ceftazidime (73.8%), cephradine (73.8%), tetracycline (69.4%), doxycycline (66.6%), augmentin (62.6%), gentamycin (59.8%), cefuroxime (58.2%), ciprofloxacin (54.2%), cefaclor (50%), aztreonam (44.8%), ceftriaxone (43.3%), imipenem (43.3%), and low level of resistance to streptomycin (30%), kanamycin (19.9%), tazocin (14%), amikacin (12.7%) and lowest to norfloxacin (11.2%). Out of 321 *E. coli* isolates, 261 (81%) were declared as multiple drug resistant and 5 (1.5%) were extensive drug resistant.

***Conclusion:*** It is concluded that most of the urinary tract infections in human are caused by multiple drug resistant *E. coli*.

## INTRODUCTION

Urinary tract infections (UTIs) are serious health affecting problems worldwide.^1^ *E. coli, E. faecalis, K. pneumoniae, S. marcescens, P. aeruginosa, S. saprophyticus, S. aureus* and *Proteus mirabilis* are most common bacteria causing UTIs in human beings.- The *E. coli* accounts for approximately 85% of community acquired UTIs and 50% of hospital acquired UTIs.^5^ Different factors like age, gender, immuno-suppression and urological instruments may affect prevalence of UTIs.^6^ Catheter-associated UTIs are one of the most dangerous health risks contributing 34% of all health care associated infections.^7^

The emergence of extended-spectrum beta-lactamases has threatened the empirical use of cephalosporins and ciprofloxacin.^8^^,^^9^ Microorganisms use various mechanisms to develop drug resistance, such as recombination of foreign DNA in bacterial chromosome, horizontal gene transfer and alteration in genetic material.^10^ Resistance pattern of microorganisms vary from country to country, state to state, large hospital to small hospital and hospital to community. In Pakistan, the problem of antibiotic resistance is compounding because of overuse and misuse of antibiotics.^6^^,^^11^ There is no systematic national surveillance of antibiotic resistance and insufficient data is available to quantify the problem.^12^ Detection of UTI causing pathogens and resistance of these pathogens to commonly prescribed antibiotics in clinical set ups is essential and helpful in improving the efficacy of empirical treatment.^13^ Objective of the present study was to highlight the bacterial etiology of UTIs and determination of resistance pattern of *E. coli* isolates.

## METHODS

The observational and prospective study was conducted at Mayo Hospital Lahore, which is one of the oldest and biggest hospitals in Punjab.

***Sample collection and Isolation of Bacteria: ***Urine samples (n=500) were collected from patients in different wards (n=400) and outpatient department (n=100) from Mayo Hospital, Lahore. Samples were centrifuged and sediments were cultured primarily on blood agar and macConkey’s agar by spread plate technique. Bacterial colonies having different morphology were selected, purified and identified by their biochemical profiles.

***Multiple drug resistance:*** Antibiotic sensitivity pattern of *E. coli* isolates was determined on Muller Hinton agar plates by Kirby-Bauer disc diffusion.^14^ Isolates were declared as sensitive or resistant on the basis of zone of inhibition following the criteria of Clinical Laboratory standards Institute.

## RESULTS

Bacterial etiology of Urinary tract infections (UTIs) in patients admitted in or visiting Mayo hospital, Lahore as out patient was determined. Resistance pattern of *Escherichia coli* against a number of antibiotics was also checked. Bacteria were successfully isolated from 402/500 samples. Rate of isolation of bacterial etiological agent from female samples (87.5%) was not-significantly higher as compared to male (71.3%) patients. Out of 402 bacterial isolates from patients, rate of *E. coli* (321, 80%) isolation was highest followed by *Staphylococcus aureus* (38 9.4%), *Proteus species (*22 5.4%) and *Pseudomonas spp* (21 5.2%). *E. coli* exhibited highest resistance to penicillin/amoxicillin (100%) followed by cefotaxime (89.7%), ceftazidime/cephradin (73.8%), tetracycline (69.4%), doxycycline (66.6%), augmentin (62.6%), gentamycin (59.8%), cefuroxime (58.2%), ciprofloxacin (54.2%), cefaclor (50%), aztreonam (44.8%), ceftriaxone/imipenem (43.3%), streptomycin (30%), kanamycin (19.9%), tazocin (14%), amikacin (12.7%) and norfloxacin (11.2%) (Table1). Out of 321 *E. coli*, 261 (81%) were multiple drug resistant and 5 isolates were extensively drug resistant. Multiple drug resistance was defined as resistance to three or more than three different antibiotic classes tested.

## DISCUSSION

UTIs are caused by microbial invasion and subsequent multiplication in urinary tract.^15^ Eighty percent of the patients with UTI had bacterial etiology in this study. Although the infection rate was higher in female (87.5%) patients as compared to male (71.3%), it was not-significant, which is in accordance with finding of Shah et al.^16^ Rate of bacterial isolation was highest in elderly patients (>50 years), which is in accordance with Iqbal et al.6 *E. coli* was observed as the most common etiologic agent of UTI, which is also in accordance with previous studies.^1^^,^^17^^,^^18^

Antibiotics are amongst the most important achievements of the twentieth century, used to kill or inhibit the growth of microorganisms. Antibiotic resistance in *E. coli* isolated from UTIs is increasing day by day, making it a major public health problem. So it is very important to determine the antibiotic resistance patterns in *E. coli* isolates for proper and accurate prescriptions.

**Table-I:** Antibiotic sensitivity pattern of *E. coli* isolates

*Antibiotics*	*Codes*	*Disks*	*Resistant*		*Intermediate*		*Sensitive*	
			*R*		*I*		*S*	
			*n*	*%*	*n*	*%*	*n*	*%*
Cephradine (CR)	CR	30 µg	237	73.8	28	8.7	56	17.4
Amikacin	AK	30 µg	41	12.7	56	17.4	224	71.7
Streptomycin	S	10 ug	96	30	76	23.6	225	70
Norfloxacin	NOR	10 ug	36	11.2	101	31.4	184	58.9
Ciprofloxacin	CIP	5 ug	174	54.2	53	16.5	94	29.2
Imipenem	IPM	10 ug	139	43.3	55	17.1	127	39.5
Cefuroxime	CXM	30 ug	187	58.2	60	18.6	74	23.0
Augmentin	AMC	30 ug	201	62.6	40	12.4	80	24.9
Ceftriaxone	CRO	30 ug	139	43.3	60	18.6	122	38.0
Gentamicin	CN	10 ug	192	59.8	44	13.7	85	26.4
Aztreonam	ATM	30 ug	144	44.8	60	18.6	177	55.1
Doxycycline	DO	30 ug	214	66.6	33	10.2	74	23
Pipracillin-Tazobactam	TZP	100/10 ug	0	0	30	9.6	291	90.6
Ceftazidime	CAZ	30 ug	237	73.8	32	9.9	52	16.1
Tetracycline	TE	30 ug	223	69.4	29	9	69	21.4
Cefaclor	CEC	30 ug	160	50	60	18.6	101	31.4
Tazocin	TZP	110/10 ug	48	14.9	0	0	273	85
Levofloxacin	LEV	5 ug	0	0	21	6.5	300	93.4
Kanamycin	K	30 ug	64	19.9	100	31.1	157	48.9
Meropenem	MEM	10 ug	0	0	0	0	321	100
Amoxicillin	AMC	20 ug	321	100	0	0	0	0
Pencillin	P	10 u	321	100	0	0	0	0
Tobramycin	TOB	10 ug	0	0	0	0	321	100
Cefotaxime	CTX	30 ug	288	89.7	0	0	33	10.2

UTIs caused by antibiotic resistant and multiple drug resistant bacteria have been increased in recent times. Complications in UTIs have increased because of the prevalence of extended spectrum beta-lactamases (ESBL) producing bacterial pathogens which are also causing many management and epidemiological issues. There were times almost a decade ago, when most of the ESBLs producing organisms were *Klebsiella* *spp.* and mostly were nosocomial. But in recent times the problem has been compounded by the prevalence of ESBL and MDR *E. coli* as well. Most of the ESBL *E. coli* are resistant to a wide range of beta lactams including cephalosporins, penicillins and piperacillin/tazobactam, and non beta lactams including fluoroquinolones, trimethoprim and gentamycin. One of the major reasons for this high resistance can be co-expressed resistance mechanisms in the species of different pathogens isolated from patients of urinary tract infections admitted to different wards of Mayo hospital Lahore, Pakistan. In the present study we analyzed their antibiotic sensitivity pattern was determined by Kirby Bauer technique.

In present study all *E. coli* species (n=321) were resistant to penicillin and amoxicillin indicating a cautious use of these antibiotics for the treatment of urinary tract infections. In different parts of the world, resistance of *E. coli* to penicillins group of antibiotics have been on higher side and is increasing day by day, but there are only few reports which indicates 100% resistance to penicillins^19^ Resistance to the combination of amoxicillin and a beta lactam inhibitor (augmentin 62.6%) was also on the higher side. Similar kinds of results, where beta lactam inhibitors increase the efficiency of penicillin group of antibiotic against *E. coli*, have been reported in previous studies.^20^ Resistance to other beta lactam antibiotics including cefotaxime (89.7%), ceftazidime (73.8%), cephradin (73.8%), cefuroxime (58.26), cefaclor (50%), Ceftrioxone (43.3%) was also very high rendering many of these inefficient for empirical prescription of these antibiotics to treat UTIs. Previous studies in Pakistan have also shown very high antibiotic resistance in *E. coli* against cephalosporins and penicillins.^21^

Generally, in developing countries like Pakistan, penicillines and cephalosporins are not active against the UTI infections and our results suggest that these antibiotics should not be used in the treatment of UTIs. Inefficiency of penicillins and cephalosporins in this study does not indicate that these antibiotics are not in use in any part of world to treat UTIs caused by *E. coli*. In some of the recent reports a higher number of *E. coli* was found sensitive to penicillins or cephalosporins from European countries.^22^ A decade before, these antibiotics were active against *E. coli* even in Pakistan.^20^

In this study, the resistance of *E. coli* against aztreonam and imipenem was 44.8% and 43.3%, respectively, which is higher than previous studies.^23^^,^^24^ Higher resistance in *E. coli* against carbapenams indicates that these may have been misused and overused in health care set ups. Tazocin, a combination of piperacillin and beta lactamases inhibitor tazobactam, showed best results, for which resistance in *E. coli* was only 14% suggesting that this antibiotic can still be used for the treatment of UTIs.^25^ Although, tetracycline group of antibiotics are not used now a days for human infectious agents, *E. coli* were highly resistant to tetracycline (69.4%) and doxycycline (67.6). In the present study variable resistance patterns were found for the aminoglycosides. *E. coli* were highly resistant to gentamycin, while low level of resistance was for kanamycin (19.9%), and amikacin (12.7%).

Quinolones, especially ciprofloxacin have been used for *E. coli* infections in recent past. In the present study however *E. coli* were highly resistant to ciprofloaxacin (54.2%), which is consistent with the previous reports.^26^ Other fluoroquinolones such as norfloxacin (11.2% resistance) and levofloaxacin (all sensitive) were found efficient for the *E. coli*. Other studies from the different parts of the world also show that quinolones are still active against UTI infections.^26^ Multiple drug resistance (MDR) and extensive drug resistance (XDR) was also determined in this study. MDR is described as resistant to at least one member from three different antibiotic classes being used for the treatment of *E. coli*, while extensive drug resistance (XDR) is described as resistance to at least one member of all but two antibiotic classes. MDR and XDR *E. coli* in this study were 81% and 8.7% respectively. The antibiotics active against the XDR were amikacin and norfloxacin generally. It is concluded that higher level of antibiotic resistance, MDR and XDR is present in *E. coli*. To treat the UTIs caused by *E. coli* combination therapy especially amikacin and ciprofloaxacin may provide better results. Antibiotic resistance in *E. coli* isolated from UTIs insinuates for its close monitoring and prescription of antibiotics after the culture sensitivity tests.

## Authors Contributions:

All the authors have contributed significantly in study design, experimentation, data analysis and manuscript drafting.
